# Intravenous enalaprilat for treatment of acute hypertensive heart failure in the emergency department

**DOI:** 10.1186/s12245-016-0125-4

**Published:** 2016-12-28

**Authors:** Syed Imran Ayaz, Craig M. Sharkey, Gregory M. Kwiatkowski, Suprat Saely Wilson, Reba S. John, Rosa Tolomello, Arushi Mahajan, Scott Millis, Phillip D. Levy

**Affiliations:** 1 Department of Emergency Medicine, Wayne State University School of Medicine, 4201 St. Antoine, UHC-6G, Detroit, MI 48201 USA; 2 Detroit Receiving Hospital, 4201 St. Antoine, Detroit, MI 48201 USA; 3 Cardiovascular Research Institute, Wayne State University School of Medicine, 540 East Canfield, Detroit, MI 48201 USA

**Keywords:** Enalaprilat, Acute heart failure, Acute hypertensive heart failure

## Abstract

**Background:**

Afterload reduction with bolus enalaprilat is used by some for management of acute hypertensive heart failure (HF) but existing data on the safety and effectiveness of this practice are limited. The purpose of this study was to evaluate the clinical effects of bolus enalaprilat when administered to patients with acute hypertensive heart failure.

**Findings:**

We performed an IRB-approved retrospective cohort study of patients who presented to the emergency department of a large urban academic hospital. Patients were identified by pharmacy record and included if they received enalaprilat intravenous (IV) bolus in the setting of acute hypertensive HF. A total of 103 patients were included. Patients were hypertensive on presentation (systolic blood pressure [SBP] = 195.2 [SD ± 32.3] mmHg) with significantly elevated mean NT-proBNP levels (3797.8 [SD ± 6523.2] pg/ml). The mean dose of enalaprilat was 1.3 [SD ± 0.7] mg, with most patients (76.7%) receiving a single 1.25 mg bolus. By 3 h post­enalaprilat, SBP had decreased substantially (−30.5 mmHg) with only 2 patients (1.9%) developing hypotension. Renal function was unaffected, with no significant change in serum creatinine by 72 h. In the 30 days post-admission, patients spent an average of 23 [SD ± 7.5] days alive and out of hospital.

**Conclusions:**

In this retrospective cohort of acute hypertensive HF patients, bolus IV enalaprilat resulted in a substantial reduction in systolic BP without adverse effect.

## Introduction

Acute heart failure (AHF) can be precipitated by multiple causes, including medication non-compliance, hypertension, acute ischemia, arrhythmias, or concurrent infection [1–3]. Regardless of the cause, patients with AHF experience an acute change in signs and symptoms requiring urgent intervention and potential hospitalization [2]. Furthermore, AHF carries significant mortality, with an estimated risk of in-hospital death of 4% [4], and morbidity, with a rate of hospital readmission of ≥ 50% in the 6 months following a first hospitalization [5–7].

Current therapies for AHF are determined based on clinical presentation [8]. Treatment guidelines from the American Heart Association recommend the use of IV loop diuretics and vasodilators to reduce pulmonary congestion [1, 3, 9]. It has been well documented that angiotensin-converting enzyme (ACE) inhibitors are effective in the treatment of hypertension and chronic HF [10–14]. Enalaprilat is a parenteral ACE Inhibitor and is the active metabolite of the orally administered pro-drug, enalapril [10, 15]. Enalaprilat has the potential to produce symptomatic improvement in AHF by its rapid hemodynamic effects, particularly when blood pressure (BP) is markedly elevated [16–18].

While afterload reduction with bolus enalaprilat is used by some for management of AHF with hypertension in the emergency department (ED), existing data on the safety and effectiveness of this practice are limited [11, 19]. However, there is real potential for delayed complications as the peak BP response may not occur for up to 4 h after initial dosing [19]. Further, excretion is biphasic with an early renal clearance (elimination half-life between 2 to 6 h) and an ensuing prolonged terminal phase (elimination half-life of 36 h) [19]. Accordingly, the purpose of this study was to evaluate the hemodynamic effects and safety of bolus enalaprilat in the treatment of patients presenting to the ED with hypertensive AHF.

## Methods

This is a retrospective cohort study of patients seen in an urban, academic, tertiary care ED in Detroit, Michigan between January 1, 2009 and July 31, 2014. Institutional Review Board approval (Wayne State University IRB reference # 091811MP2E) was obtained before abstracting data. The study cohort consisted of all patients greater than 18 years old, who presented to the ED with hypertensive AHF. Patients were identified by pharmacy record review of those who received enalaprilat by IV bolus in the ED. Electronic medical records for each identified patient were then reviewed, and only those for whom IV enalaprilat was used to treat AHF were included. Eligible patients could have received one or more intermittent doses of IV enalaprilat, with or without additional diuretic or vasodilator therapy, such as continuous nitroglycerin infusion or IV loop diuretics.

Baseline data were compiled along with hemodynamic response over time, and the following outcome measures were tracked: in-hospital mortality, admission location, length of hospital stay, and final hospital disposition. The study also analyzed the rate of endotracheal intubation and non-invasive positive pressure ventilation (NIPPV) initiated in the ED, and bolus doses of IV loop diuretics administered in the ED. Furthermore, the study analyzed the effects of IV enalaprilat on renal and cardiac perfusion, measured from the estimated glomerular filtration rate (eGFR) and elevation in cardiac troponin, respectively. Serum creatinine and eGFR were recorded on initial presentation to the ED and again daily for the first 3 days of admission, if available. Cardiac specific troponin I was recorded on presentation to the ED, 6–8 h after presentation, and 24 h after presentation, if available. All troponin samples were analyzed using a Siemens® Dimension EXL chemistry analyzer, and a value of ≥ 0.20 ng/mL was considered positive. The effect of IV enalaprilat on BP was assessed on presentation to the ED and again at 30 min, 1 h, 2 h, and 3 h post-enalaprilat administration, if available. Final disposition, such as in-hospital mortality and discharge locations, were recorded on discharge from the hospital. Readmissions to the hospital and ED revisits in the 30-day period post-discharge were assessed using the electronic medical records. The incidences of ACE inhibitor-induced angioedema were also evaluated.

Data were analyzed with Stata 14 using maximum likelihood for parameter estimation. We used a mixed effects modeling approach to examine change in systolic blood pressure over time. We hypothesized that, for each individual, blood pressure would be a specified function of time along with error. This trajectory is commonly expressed as a linear function of time containing two unknown individual latent growth factors: an intercept and a slope. The individual intercept parameter represented mean number blood pressure at 30 min post-drug administration. The individual slope parameter represented the rate of change in blood pressure over time, up to 180 min post-drug administration. Since the number of observations per individual was relative, we employed a linear individual growth model, rather than including a cubic or quadratic term. After an individual growth trajectory model is specified at Level-1 to represent the individual change over time, a Level-2 model can be specified in which covariates or predictor variables thought to be related to or hypothesized to affect the individual growth parameters are entered into the model. Hospital and intensive care unit length of stay and IV enalaprilat doses were compared using an unpaired *t* test. Rates of endotracheal intubation and NIPPV were compared using Fisher’s exact test. The study was approved by the Wayne State University Human Investigations Committee prior to initiation. All data were abstracted according to previously published guidelines by Gilbert and colleagues [20] using a standardized data collection form and specifically trained chart abstractors.

## Findings

### Demographics

A total of 103 patients were included, all of whom received at least one dose of bolus enalaprilat. The mean age was 63 years (SD± 14) with 61% male and 96% African Americans. Tables 1 and 2 show medical history and home medications at the time of ED presentation.

**Table 1:** Past medical history

Medical history	% of patients
Alcoholism	0.9%
Cocaine use	10.6%
Tobacco smoking	17.4%
Intravenous drug user	5.8%
Diabetes mellitus type II	27.1%
Chronic obstructive pulmonary disease	16.5%
Asthma	12.6%
Chronic kidney disease	15.5%
End stage renal disease (on hemodialysis)	5.8%
Known history of heart failure	59.2%
Hypertension	82.5%
Atrial fibrillation	5.8%
Myocardial infarction	3.8%
Coronary artery disease	5.8%

**Table 2:** Home medications

Medications	% of patients
ACE Inhibitor	34.9%
Beta blocker	49.5%
Aspirin	29.1%
Loop diuretic	41.7%
Non-loop diuretic	7.7%
Potassium-sparing diuretic	6.7%
Digoxin	3.8%
Isosorbide mononitrate	12.6%
Hydralazine	15.5%

### ED course and treatment

Patients were markedly hypertensive on presentation (systolic blood pressure = 195.2 [SD ± 32.3] mmHg) with significantly elevated NT-proBNP levels (3797.8 [SD ± 6523.2] pg/ml). The mean left ventricular ejection fraction (LVEF) was 38% (SD ± 19). 88.3% of patients received IV furosemide with a mean dose of 64.1 (SD ± 29.5) mg. High dose nitroglycerin (2 mg IV bolus) was given in 13.5% of the patients. A second dose of 2 mg IV bolus was given to 6.7% of the patients, with only 1 patient (0.97%) receiving a third dose of 2 mg IV bolus. Continuous nitroglycerin IV infusion was started in 21.3% of patients, with 18.4% receiving an initial drip at 50 mcg/min, and 2.9% receiving 100 mcg/min.

**Fig. 1:**
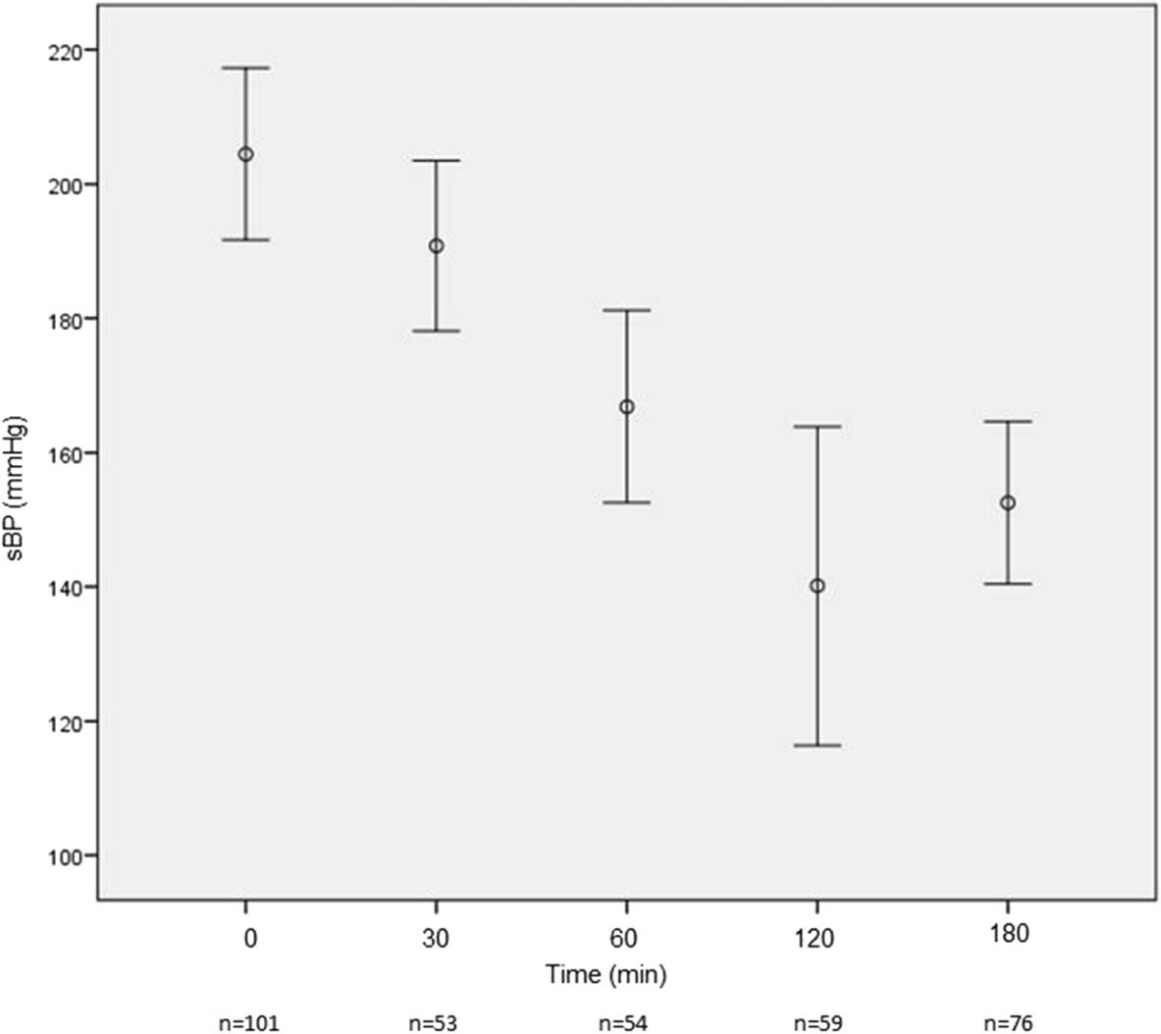
Systolic blood pressure at baseline and after enalaprilat administration

**Fig. 2:**
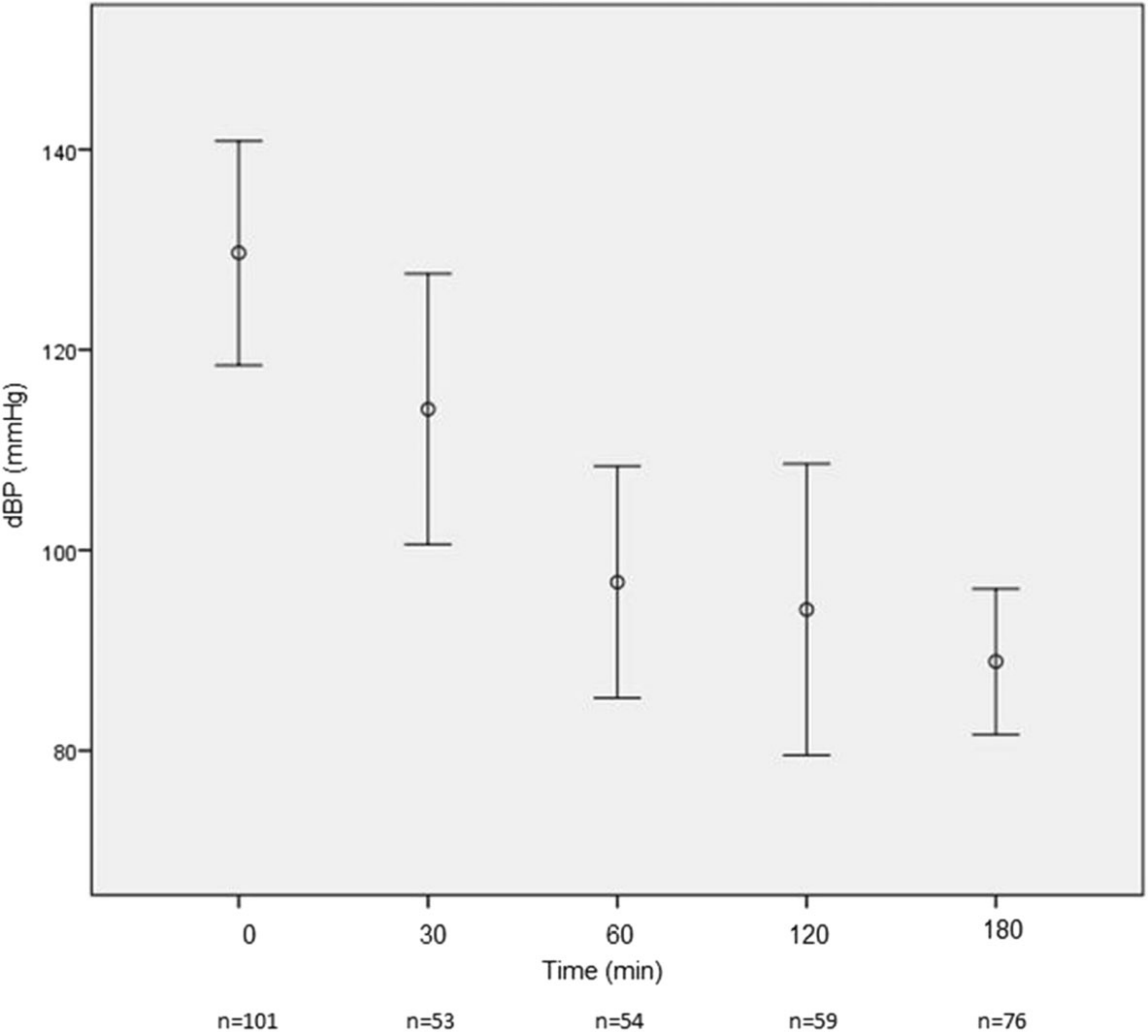
Diastolic blood pressure at baseline and after enalaprilat administration

**Fig. 3:**
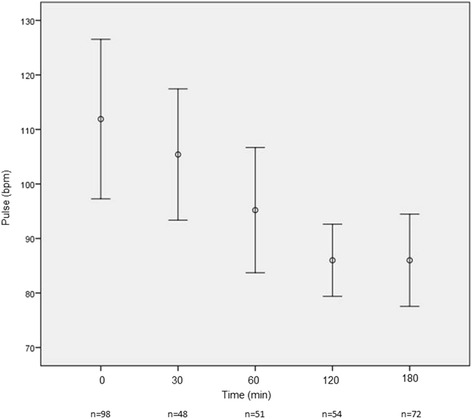
Heart rate at baseline and after enalaprilat administration

**Fig. 4:**
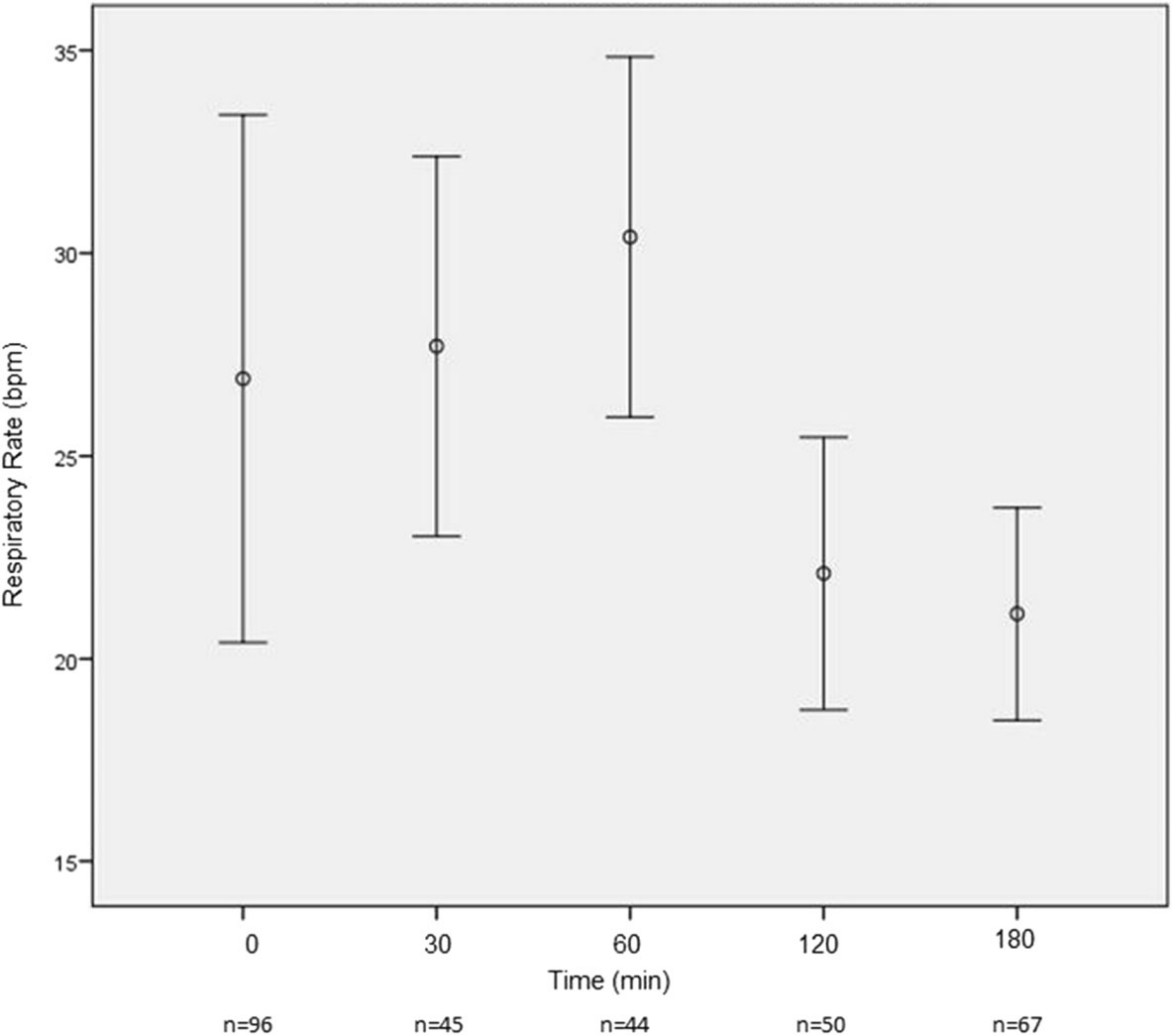
Respiratory rate at baseline and after enalaprilat administration

**Fig. 5:**
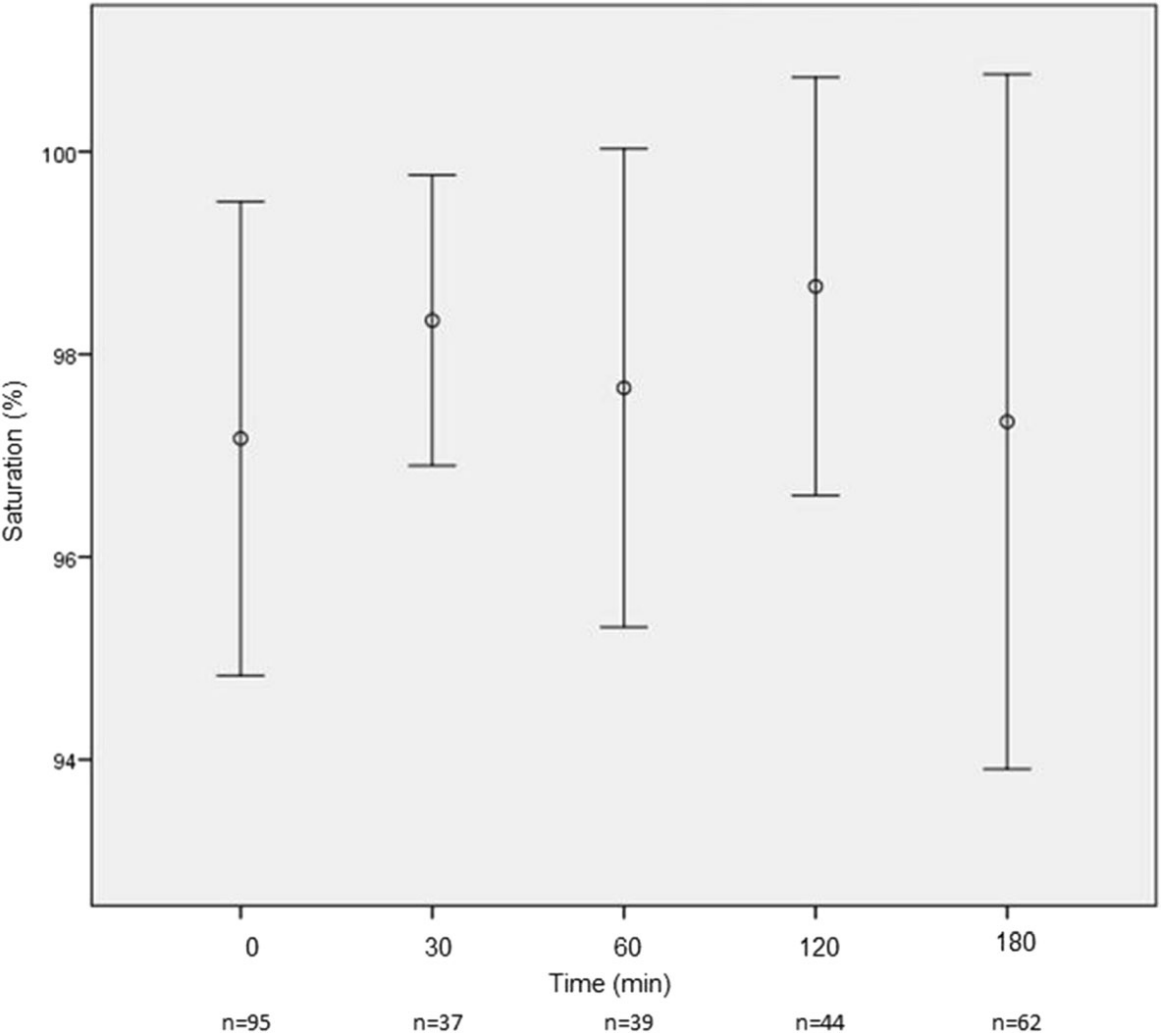
Pulse oximetry at baseline and after enalaprilat administration

The mean time to enalaprilat administration in the ED after presentation was 121.5 min (SD ± 144.9). The mean dose of enalaprilat was 1.3 (SD ± 0.7) mg, with most patients (77.7%) receiving a single 1.25 mg bolus. By 3 h post­enalaprilat, systolic blood pressure (BP) had decreased substantially (−30.5 mmHg) with only 2 patients (1.9%) developing hypotension (systolic BP < 90 mmHg). Figures 1, 2, 3, 4, 5 depict vital signs at baseline and 30, 60, 120, and 180 min after enalaprilat administration.

In our initial model, only the intercept was allowed to vary. The fixed effect of time was statistically significant, *p* = 0.0001: SBP decreased by a mean of 8.67 mmHg over each of the four time periods. In addition, the intercept contained significant variability (*p* = 0.0001) available for prediction in the Level-2 model. The second model, in which both the intercept and slope were allowed to vary, did not provide improved model fit compared to the simpler intercept-only model. In other words, there was evidence that participants showed significant variability at the beginning of treatment but reduction in SBP over time was consistent across people. We then attempted to explain changes in SBP by entering explanatory covariates into our model: time, age, gender, admission SBP, admission GFR, and total amount of enalaprilat given. As in our intercept-only model, time was statistically significant. Admission SBP was also significant, which is not surprising: the higher the admission SBP, the higher it was over time. The remaining covariates were not significant, including the total amount of enalaprilat given. However, there was limited explanatory variance in this variable, i.e., over 75% of participants were given the same dose of the medication.

We also used a mixed effects model with a random intercept to examine the impact of enalaprilat plus nitroglycerin versus enalaprilat alone on SBP. This group variable was entered into the model while controlling for age, gender, admission SBP, and admission GFR. The time/period effect was significant with an average reduction in SBP of over 8 mmHg with each period. As with our other models, admission SBP remained significant. While there was not a statistically significant difference/effect of added nitroglycerin (*p* = 0.18), patients who received nitroglycerin had about a 7-point lower SBP than those who received enalaprilat alone.

Most (80.6%) patients had a troponin concentration obtained during their initial ED admission, which was positive in 58.1%. Fewer patients had a troponin drawn at 6–8 h (59.2%) or 24 h (45.6%) post-admission, only 9% of which were positive. Serum BUN, creatinine, and glomerular filtration rate were largely unaffected with no significant change by 72 h (Table 3). Only 7.8% were placed on NIPPV and 3.9% required endotracheal intubation. No patient developed angioedema during or post-treatment.

**Table 3:** Renal function at presentation and during index hospitalization

	ED presentation	Day 1	Day 2	Day 3
	*n* = 103	*n* = 83	*n* = 70	*n* = 64
Serum BUN (mg/dL)	23.7 [SD ± 14.1]	25.7 [SD ± 16.4]	26.2 [SD ± 14.1]	28.0 [SD ± 16.8]
Serum creatinine (mg/dL)	2.39 [SD ± 4.35]	2.16 [SD ± 2.0]	2.0 [SD ± 1.5]	2.2 [SD ± 1.9]
GFR (mL/min/1.73 m^2^)	59.5 [SD ± 30.3]	59.2 [SD ± 30.0]	59.0 [SD ± 29.0]	57.2 [SD ± 31.9]

*SD* standard deviation; *BUN* blood urea nitrogen; *GFR* glomerular filtration rate

### Disposition

Nearly one third (30.1%) of patients were admitted to the ICU; 27.2% were admitted to a telemetry floor bed, 35.0% were admitted to observation unit, and 7.7% admitted to the regular medical floor. The mean (SD) length of hospital stay was 4.6 (4.1) days, with a mean standard deviation ICU length of stay of 2.6 (1.7) days. After the hospital stay, the majority of patients (85.7%) were discharged home; 7 patients were sent to long-term care facilities, and 2 were sent to rehabilitation centers. There were no patients who died in hospital. In the 30 days after admission, patients spent an average of 23 (SD ± 7.5) days out of hospital and alive.

## Discussion

Data from our retrospective cohort show a substantial decrease in SBP (−30.5 mmHg) within 3 h post­enalaprilat administration. Although relatively small, evidence from previous clinical trials suggests that enalaprilat is generally well tolerated [11]. Our findings support this, with no evidence of adverse effects on renal function or cardiac perfusion, and no evidence of excess in-hospital mortality or post-discharge recidivism. The most plausible concern with an IV bolus administration of enalaprilat is hypotension [11]. However, we found few episodes of hypotension in our patient population suggesting that IV enalaprilat is safe for administration in the setting of hypertensive AHF. Additionally, while 96% of our study cohort was African American, no patients experienced ACE inhibitor-induced angioedema, suggesting that any concerns for acute onset of this well-known side effect are unfounded.

Traditionally IV diuretics have been used in the early management of patients presenting with AHF in the ED [11]. However, the latest evidence suggests that there is a particular phenotype of HF patients that present to the ED with acute hypertension and can be managed more appropriately with IV vasodilators as an adjunct to diuretic therapy [21–24]. The American Heart Association, in its 2013 practice guidelines, recommended nitrovasodilators and nesiritide in hospitalized hypertensive HF patients (class IIb, level of evidence A) [3]. Although IV vasodilators are increasingly being used in the ED setting, a recent systematic review of existing literature found a lack of evidence and clinical experience for the use IV enalaprilat in ED patients with acute hypertensive HF. [11] Our data add to the existing evidence base, providing treatment response and outcome data for the largest cohort to date who were treated with IV enalaprilat in the setting of hypertensive AHF.

### Limitations

The primary limitation of this study was its retrospective nature. While this precludes the ability to capture relevant clinical information on all patients in a uniform manner, we were able to compile outcome data on all subjects and repeat measure data on a reasonable proportion, thus yielding sufficient information to base effect size estimation for potential future studies. As well, the sample size itself is relatively small; however, this reflects the pragmatic design of our study and all patients who received IV enalaprilat during the study period were included. Although the number of patients at our institution who received IV enalaprilat may seem low (approximately 20 patients per year over the study period), our approach to case ascertainment using pharmacy records makes it unlikely that we missed any patients who were treated in this manner. Symptom (dyspnea) relief is an important outcome to report in any study investigating AHF treatment in the ED. However, time to symptom relief is not documented consistently in patients’ charts (electronic medical records) for accurate retrospective analysis. In addition, patients did receive other concurrent antihypertensive therapy which could have confounded noted effects on BP and other measures. Future studies are clearly warranted to see if enalaprilat offers any specific advantages over other interventions, and our data has utility for the design of such trials.

## Conclusions

In this retrospective cohort study, use of bolus IV enalaprilat was well tolerated, resulting in a substantial reduction in systolic BP with limited hypotension and no adverse effect on renal function when administered to hypertensive patients with AHF. While prospective comparison trials are needed, these data suggest a potential role for bolus IV enalaprilat in the management of hypertensive AHF.

## Abbreviations

ACE: Angiotensin-converting enzyme
AHF: Acute heart failure
BP: Blood pressure
ED: Emergency department
GFR: Glomerular filtration rate
HF: Heart failure
IV: Intravenous
LVEF: Left ventricular ejection fraction
NIPPV: Non-invasive positive pressure ventilation
NT-proBNP: N-terminal pro b-type natriuretic peptide
SD: Standard deviation
